# Machine-learning-based diagnosis of thyroid fine-needle aspiration biopsy synergistically by Papanicolaou staining and refractive index distribution

**DOI:** 10.1038/s41598-023-36951-2

**Published:** 2023-06-17

**Authors:** Young Ki Lee, Dongmin Ryu, Seungwoo Kim, Juyeon Park, Seog Yun Park, Donghun Ryu, Hayoung Lee, Sungbin Lim, Hyun-Seok Min, YongKeun Park, Eun Kyung Lee

**Affiliations:** 1 Division of Endocrinology and Metabolism, Department of Internal Medicine, National Cancer Center, Goyang, 10408 South Korea; 2 Tomocube Inc., Daejeon, 34051 South Korea; 3 Artificial Intelligence Graduate School, Ulsan National Institute of Science and Technology (UNIST), Ulsan, 44919 South Korea; 4 Department of Physics, Korea Advanced Institute of Science and Technology (KAIST), Daejeon, 34141 South Korea; 5 KAIST Institute for Health Science and Technology, KAIST, Daejeon, 34141 South Korea; 6 Deparment of Pathology, National Cancer Center, Goyang, 10408 South Korea; 7 Present Address: Department of Electrical Engineering and Computer Science (EECS), MIT, Cambridge, MA 02139 USA; 8 Department of Statistics, Korea University, Seoul, 02841 South Korea

**Keywords:** Cancer, Endocrine cancer, Optics and photonics, Optical techniques

## Abstract

We developed a machine learning algorithm (MLA) that can classify human thyroid cell clusters by exploiting both Papanicolaou staining and intrinsic refractive index (RI) as correlative imaging contrasts and evaluated the effects of this combination on diagnostic performance. Thyroid fine-needle aspiration biopsy (FNAB) specimens were analyzed using correlative optical diffraction tomography, which can simultaneously measure both, the color brightfield of Papanicolaou staining and three-dimensional RI distribution. The MLA was designed to classify benign and malignant cell clusters using color images, RI images, or both. We included 1535 thyroid cell clusters (benign: malignancy = 1128:407) from 124 patients. Accuracies of MLA classifiers using color images, RI images, and both were 98.0%, 98.0%, and 100%, respectively. As information for classification, the nucleus size was mainly used in the color image; however, detailed morphological information of the nucleus was also used in the RI image. We demonstrate that the present MLA and correlative FNAB imaging approach has the potential for diagnosing thyroid cancer, and complementary information from color and RI images can improve the performance of the MLA.

## Introduction

Thyroid nodules are common in the general population, and some advance to thyroid cancers that require surgery^1^. Thyroid fine-needle aspiration biopsy (FNAB) is the most important preoperative diagnostic modality for distinguishing between benign and malignant thyroid nodules^1^. The detection of thyroid nodules and the frequency of thyroid FNAB has increased significantly worldwide with the increasing utilization of diagnostic imaging modalities^2–5^. Evaluation of FNABs is still hindered by multiple challenges, including dependence on highly skilled cytopathologists, and interobserver variability, which is further complicated by the quality of image data presented for interpretation.

Recently, machine learning algorithms (MLAs) are increasingly being applied to medical imaging and tumor pathology and are expected to become a promising tool that can help reduce the time required for diagnoses by experts or increase the diagnostic accuracy of thyroid FNAB^6,7^. Recently, MLAs have shown high overall accuracy in diagnosing thyroid cancer using digital imaging of thyroid FNAB specimens^8^ and have well distinguished benign and malignant nodules from the indeterminate ones, with surgically proven pathological diagnosis^9,10^. Nevertheless, MLA-based diagnostic tools for thyroid FNAB have not yet been commercialized, with further studies being required before they can be applied in the clinical field^11^.

Interestingly, the recent improvements in the performance of MLAs have advanced algorithms for thyroid FNAB, making it possible to classify the given digital medical imaging data more effectively^7,8,12,13^. However, the method for acquiring digital data and the retrieval of imaging information to be utilized for MLAs from thyroid FNAB specimens has been poorly studied and neglected, despite being an important determinant of MLA performance. Most previous studies have used color monolayer images of Papanicolaou-, Giemsa-, and hematoxylin–eosin-stained specimens or morphometric parameters calculated from these images^7,8,13^. The advantage of using these images is that they are relatively easy to obtain, clinicians are familiar with them, and they represent the current standard of practice. However, whether these are the best digital data for accurately diagnosing thyroid cancer through MLA remains unclear.

To maximize the advantages of MLAs, high-quality, high-resolution, and high-content images are required^14,15^. This is a prerequisite for correctly assessing the characteristics of suspicious FNABs. The aim of this study was to pursue a higher content of cytopathology end-points and evaluate the potential of diagnoses using standard thyroid FNAB brightfield microscopy images combined with an emerging quantitative phase imaging technique (QPI). QPI exploits the intrinsic refractive index (RI) distribution of cells and tissues as quantitative label-free imaging contrast^16,17^. RI images can show complementary and synergistic features to brightfield microscope-based color images for the same cells or tissues due to the differences in imaging methods^18^. RI images provide structural or morphological information of cellular or subcellular structures^17,19–21^, whereas brightfield images of Papanicolau-stained slides provide molecular-specific information^22^. More importantly, RI is a quantitative and reproducible quantity; it is a physical feature that remains constant regardless of the venue from where it is obtained. Therefore, obtaining high quality images less dependent on sample preparation and working^23–25^. In this study, we trained and tested an MLA to distinguish between benign and malignant thyroid cell clusters using digital color- and RI-images of Papanicolaou-stained thyroid FNAB specimens. Furthermore, we investigated whether the information from RI images could improve the accuracy of the MLA by supplementing information from color images for the same specimens.

## Materials and methods

### Thyroid cell cluster specimens

We performed a single-center cross-sectional study of thyroid cell clusters obtained via thyroid FNAB from benign or malignant human thyroid nodules. Thyroid FNAB slides produced from July 1, 2020, to December 31, 2020, were selected from the medical database of the institution. A benign case was defined as a case in which the FNAB result was “benign (II)” according to The Bethesda System for Reporting Thyroid Cytopathology (TBSRTC)^26^. A malignant case was defined as a case in which the FNAB result was “suspicious for malignancy (TBSRTC V)” and was confirmed to be papillary thyroid carcinoma using surgical specimens or the result was “malignant (TBSRTC VI)”. One Papanicolaou-stained liquid-based cytology smear slide per patient was selected. An expert pathologist reviewed each slide and randomly selected up to 20 thyroid cell clusters per slide. Cell clusters were excluded when (a) they originated from thyroid cancer but did not contain cells with characteristics of malignancy or (b) the quality of digital images obtained from them was insufficient for analyses.

### Image acquisition and processing

**Figure 1:**
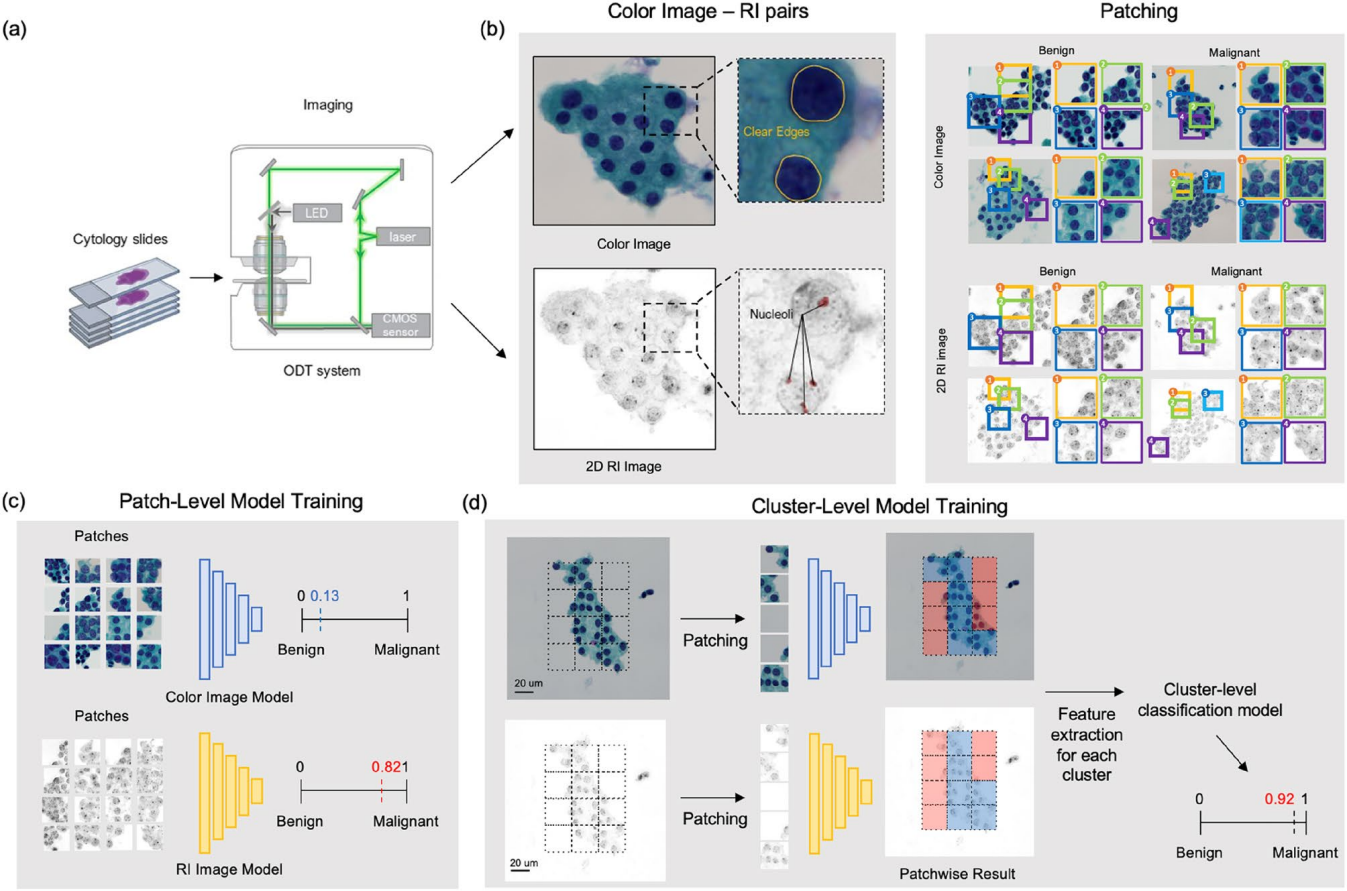
Overall scheme of the research. (**a**) Two types of images (color and RI images) of thyroid cell clusters were taken from cytology slides using the ODT system equipped with a brightfield microscope. (**b**) Each cluster image was then patched into 256 × 256-pixel patches to train the patch-level malignancy classification model. (**c**) The resulting patch-level classification models were used to generate a malignancy prediction map for each cluster to extract features to train the cluster-level model. (**d**) The resulting model outputs the malignancy of the cluster.

For each thyroid cell cluster, one two-dimensional color photograph and one three-dimensional RI tomograph were simultaneously acquired using the optical diffraction tomography (ODT) system equipped with a brightfield imaging acquisition module. For this study, we built a correlative ODT system by modifying an existing ODT system (HT-2H, Tomocube Inc., Daejeon, Republic of Korea) (Fig. 1a). Three-dimensional RI tomograms were then converted into two-dimensional RI images by projection along the Z-axis, to synchronize the model structure with that of color images.

Due to the varying sizes of thyroid cell clusters, using the predicted information from the fixed-size small regions of interest (patches) extracted from the images of clusters is more efficient. Therefore, each image containing a cluster was divided into numerous 256 × 256-pixel (26.1 μm × 26.1 μm) patches. Each patch overlapped adjacent patches by 128 pixels in one direction. The average count of the color image value was calculated for each patch, and we found that the patch with average counts of color image ≥ 170 generally contained a whole or a part of clusters within the patch. These patches were used as the smallest unit for analysis in this study; the patches with an average count of color image ≥ 170 containing only background materials were included without manual exclusion to increase generality.

### MLA training and testing

The cluster and patch images were divided into training, validation, and test datasets for the deep learning models with respect to the ratio of malignancy over dataset. Images generated from one cluster were categorized together while dividing the dataset (i.e. patch images from the same cluster were included in either training, validation, or test datasets in batches).

The architecture of the MLA comprised two levels: patch-level and cluster-level (Fig. 1b). The detailed structure of the system is described separately (Supplementary Text 1, 2). Briefly, we first trained the MLA for patches in the CNN architecture (DenseNet-169) on a binary classification task to identify patches extracted from malignant cell clusters. Color images and RI images were used separately to generate two patch-level MLAs (color-model and RI-model) (Fig. 1c). Consequently, the trained patch-level classification model generated a malignancy prediction heatmap for each cluster. The features of each cell cluster were extracted based on the heatmap, and a final tree-based cluster-level classification model XGBoost classifier was trained using these features (Fig. 1d). MLA models were generated using only color images (color-model), only RI images (RI-model), or both the types of images together (combined model), and their diagnostic performance was evaluated and compared based on the sensitivity, specificity, positive predictive value (PPV), negative predictive value (NPV), and accuracy.

### Explanatory analysis

Details of the explanatory analyzes are described separately (Supplementary Text 3, 4). Briefly, we used gradient-weighted class activation mapping (Grad-CAM) to interpret the MLA classification process. Grad-CAM emphasized the local features of the points wherein MLA judges malignancy. Additionally, patch images were grouped based on the prediction score of patch-level MLA (i.e., how highly the MLA judged the probability that a given patch was extracted from a malignant cluster) or using t-distributed stochastic neighbor embedding (t-SNE) analysis. In each group, the sizes of the nucleus and the degree of detail of the images around the nucleus were evaluated. The degree of detail of the images was quantitatively evaluated using the Brenner gradient.

### Ethics statement

This study was conducted according to the guidelines of the Declaration of Helsinki and was approved by the institutional review board of the National Cancer Center (IRB number: NCC2020-0126), which waived the requirement for informed consent for this study.

## Results

### Patients and specimens

Overall, 1,535 thyroid cell clusters obtained from 124 patients were included in this study (Table 1, and Supplementary Table 1). The numbers of benign and malignant clusters were 1,128 (73.5%) and 407 (26.5%), respectively. Cell clusters were divided into training (n = 988), validation (n = 261), and test (n = 286) datasets, and the ratio of the benign and malignant clusters in each dataset was maintained similar to the ratio in the entire dataset.

**Table 1:** Dataset description.

	Benign		Malignancy		Total	
	Clusters	Patches	Clusters	Patches	Clusters	Patches
Train set	721 (73.0%)	9315 (63.0%)	267 (27.0%)	5480 (37.0%)	988	14,795
Validation set	191 (73.2%)	2312 (57.3%)	70 (26.8%)	1721 (42.7%)	261	4033
Test set	216 (75.5%)	2888 (63.8%)	70 (24.5%)	1642 (36.2%)	286	4530
Total	1128 (73.5%)	14,515 (62.1%)	407 (26.5%)	8843 (27.9%)	1535	23,358

The number of clusters and patches for the training, validation, and test dataset. The dataset is categorized with respect to the ratio of benign and malignant clusters while maintaining the overall class ratio. The splitting ratio of training, validation and test dataset of patch dataset is 0.64:0.17:0.18, where the ratio of cluster dataset is 0.63:0.17:0.19.

### Model performance

Due to the training of the patch-level classification model, the color-model showed an accuracy of 0.975, which was better than that of the RI-model (0.937) (Table 2 and Supplementary Fig. 1). False negatives accounted for a considerable amount (3.75% of the total count) of the overall classification results of the patch-level RI-model. Most false-negatives were accounted for patches with noise or artifacts caused by the staining process.

**Table 2:** Results table.

		Prediction					Confusion matrix			
		Accuracy	Sensitivity	Specificity	PPV	NPV	True negative	False positive	False negative	True positive
Patch-level classification	Color	0.975	0.969	0.978	0.962	0.983	2838 (62.6%)	63 (1.39%)	50 (1.10%)	1579 (34.9%)
	2D RI	0.937	0.900	0.959	0.929	0.941	2718 (60.0%)	117 (2.58%)	170 (3.75%)	1525 (33.7%)
Cluster-level classification	Color	0.980	0.929	1.0	1.0	0.980	181 (72.1%)	0 (0%)	5 (1.99%)	65 (25.9%)
	2D RI	0.980	0.957	0.989	0.971	0.980	179 (71.3%)	2 (0.80%)	3 (1.20%)	67 (26.7%)
	Combined	1.0	1.0	1.0	1.0	1.0	181 (72.1%)	0 (0%)	0 (0%)	70 (27.9%)

The performance of algorithms for patch-level and cluster-level classification is presented.

RI, refractive index; PPV, positive predictive value; NPV, negative predictive value. Clusters that have less than five patches were excluded in the result table.

In the cluster-level classification, the combined model using information from both types of images showed an accuracy of 1.000 (perfect classification of benign and malignant clusters), which was higher than that of the models using only a single imaging modality (0.980 for the color-model, and 0.980 for the RI-model).

We also conduct experiments on different MLA models on cluster-level classification including Random Forest, Support Vector Machine and Multi-layer Perceptron. We can confirm the robustness of performances on different models and the relationship between color and RI images. The model performances are summarized in Supplementary Table 3.

### Gradient-weighted class activation mapping

**Figure 2:**
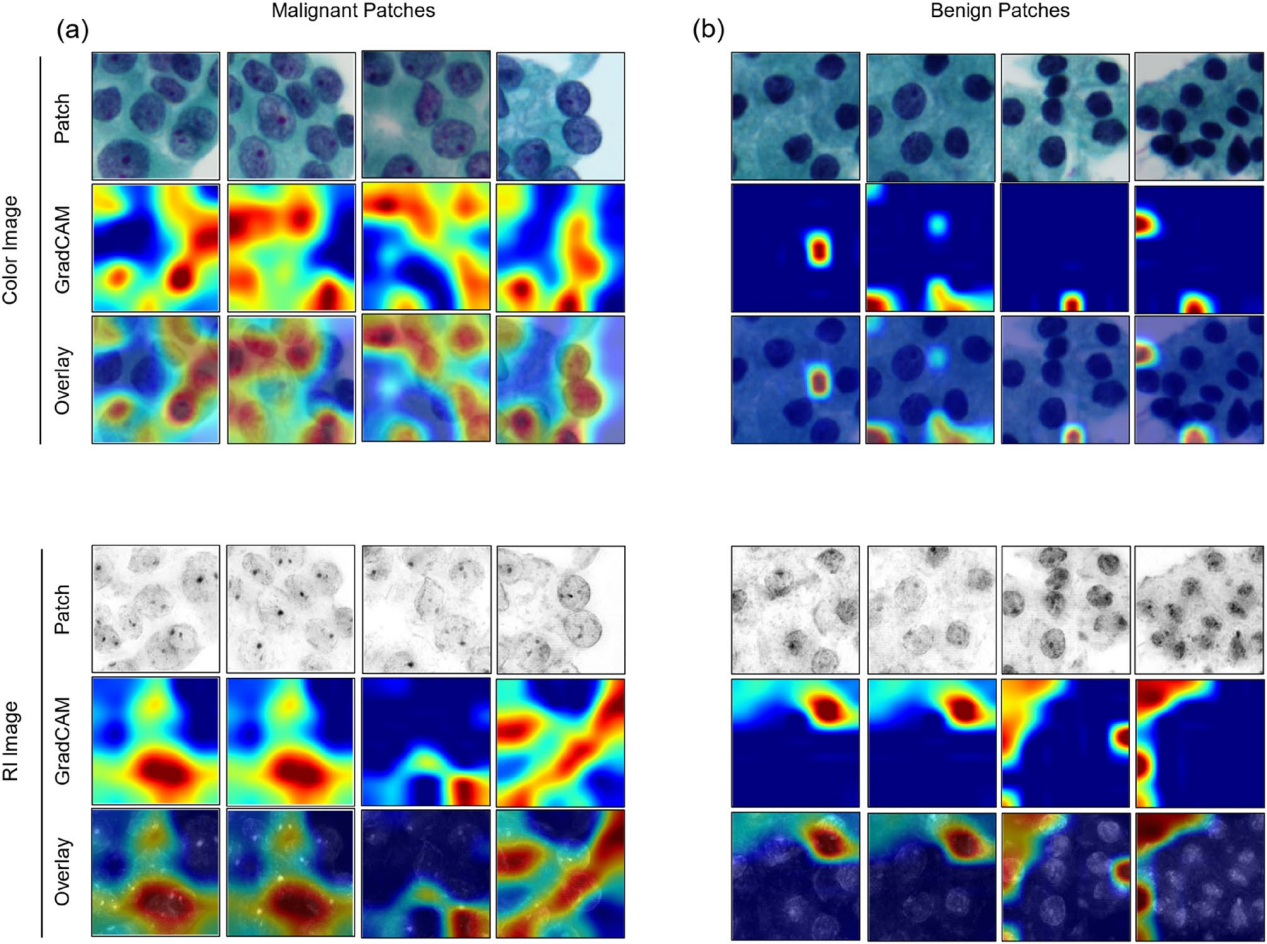
GradCAM of the patch-level classification model. Patch-level model visualizations of (**a**) malignant and (**b**) benign patches, using GradCAM for color patch images and 2D RI patch images. Red color in the GradCAM indicates a high gradient. Different peaks in the GradCAM of the color patch model and the 2D RI patch model show that the two models focus on different features of the cellular images.

Grad-CAM results of the selected patches are presented in Fig. 2. The overlay image showed that the color-model and RI-model focused on distinct areas for the same specimen. Activation of the color-model mainly appeared in large-sized nuclei, indicating that a patch is highly likely to be classified as a malignancy in the presence of large-sized nuclei. In contrast, the RI-model showed high activation in the nuclei with high image gradients and relatively clear intranuclear structures.

### Image characteristics according to model prediction scores

**Figure 3:**
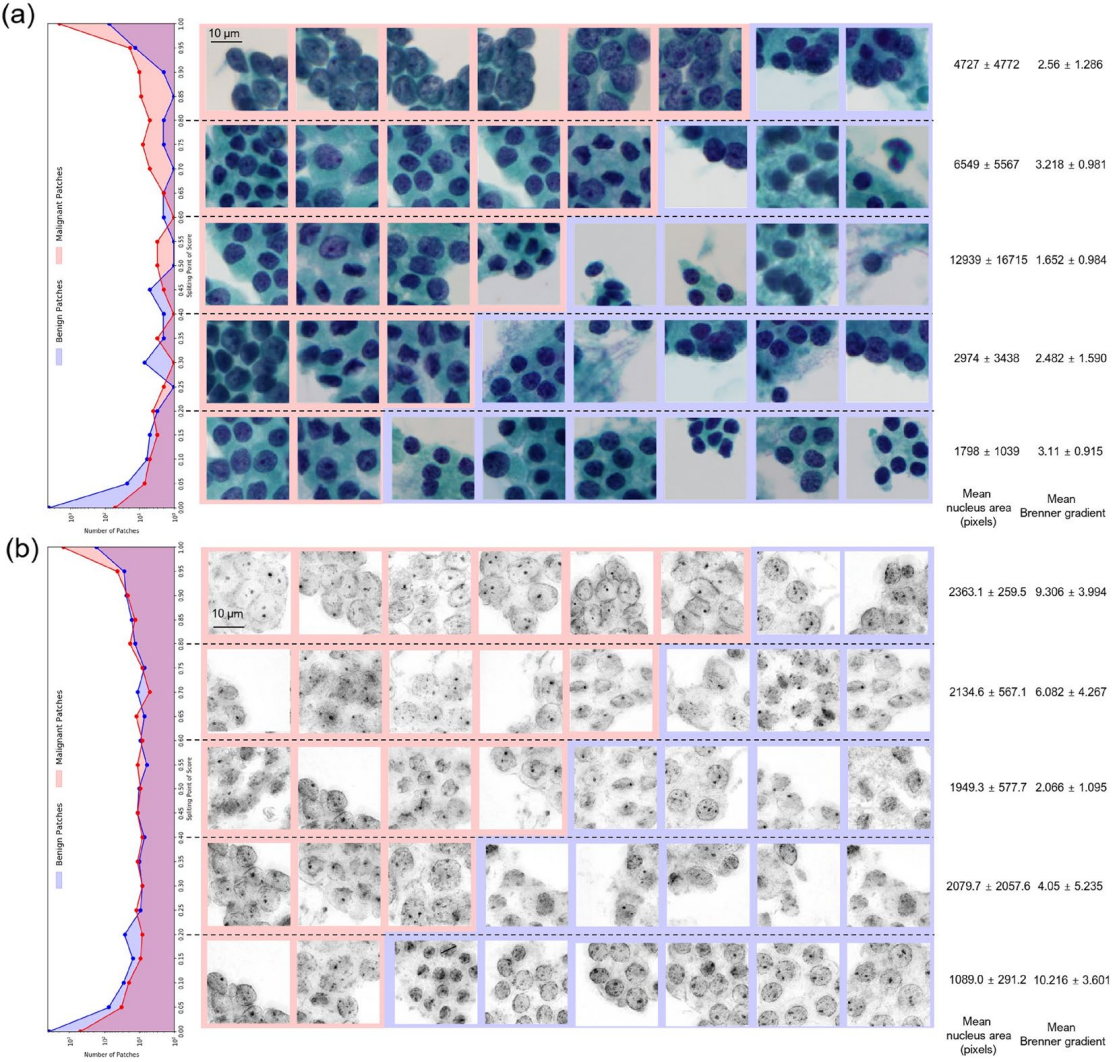
Representative patches with different prediction scores. Frequency histograms of the classification scores for (**a**) the color image and (**b**) 2D RI image patches from 0 to 1 with an interval of 0.05. In the five groups classified using 0.2 point-intervals of the classification scores, the representative images, mean nuclear area, and mean Brenner gradient are presented. The corresponding red and blue boxes are patches from the malignant and benign clusters, respectively. The mean nucleus area and mean Brenner gradient were calculated using 30 randomly chosen samples for each interval.

Patch images according to the prediction score of the patch-level model were visualized and analyzed to demonstrate the differences in trends between the color-model and RI-model (Fig. 3). The correlation between the size of the nuclei and the prediction score was prominent in the color-model (the larger the nucleus, higher the probability of malignancy) but was less pronounced in the RI-model.

The degree of details of the images surrounding the nuclei quantified using the Brenner gradient was high when the prediction score of the RI-model was either very high (0.8–1.0) or very low (0.0–0.2), whereas the model confidence was high. This finding indicates that the more detailed the shape around the nucleus, the more clearly the cells could be distinguished, whether benign or malignant and that the RI-model performed classification by focusing on the detailed structure of the nuclei. In contrast, the relationship between the prediction score and Brenner gradient was not obvious for the color-model.

### T-distributed stochastic neighbor embedding analysis

**Figure 4:**
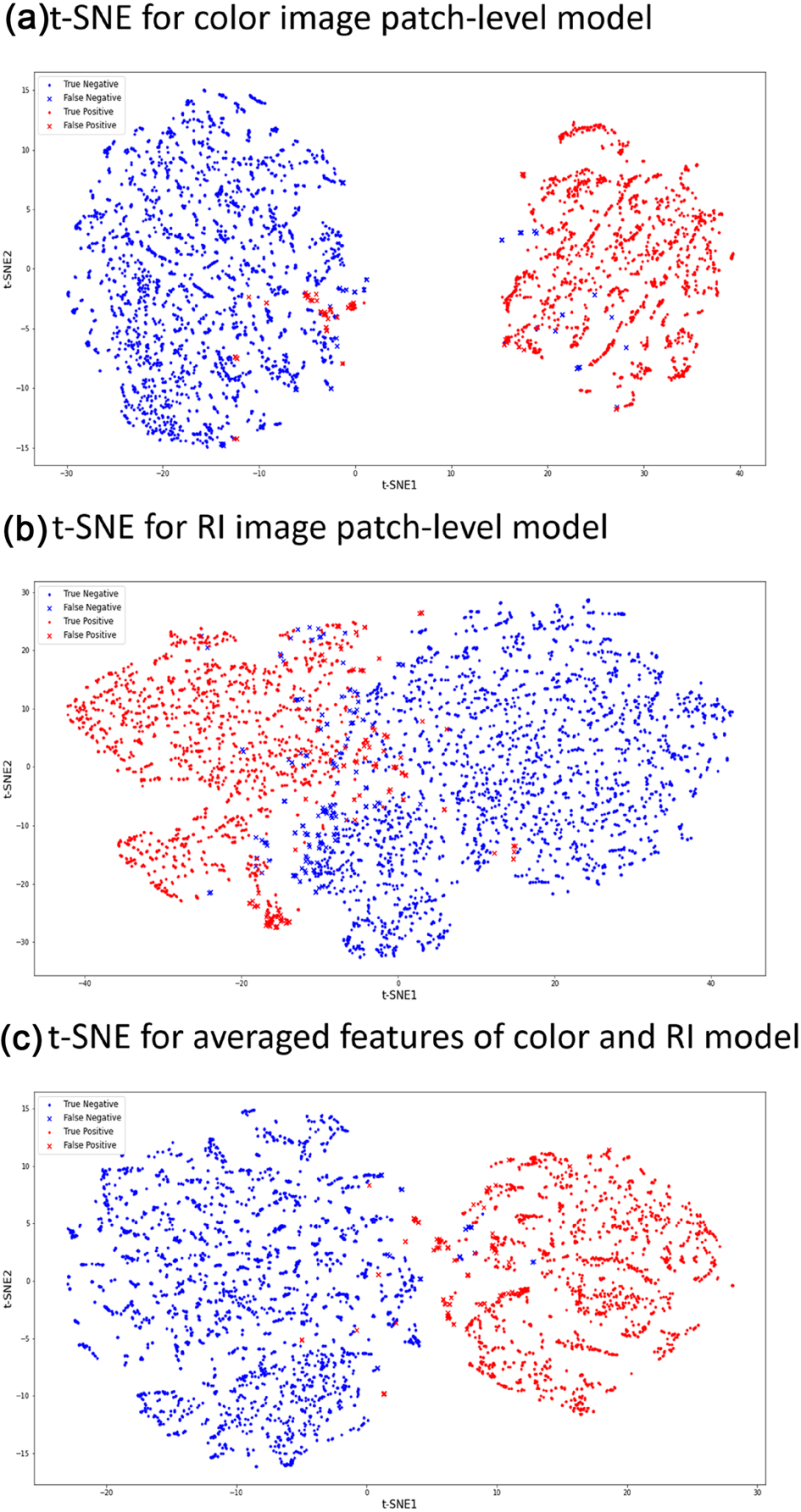
tSNE analysis: t-SNE analysis of Papanicolaou-stain and the RI patch-level model. (**a**) Papanicolaou stain patches showed distinct grouping. Each group showed different sizes and shapes of the nucleus. (**b**) RI patches showed relatively weak grouping while still showing a difference in nucleus size and shape for each group. In the grey area of the tSNE map, observing detailed structures in the RI patch was difficult. (**c**) When both the Papanicolaou staining and RI images were used together, the benign and malignant groups could be more distinctly separated.

t-SNE analysis was performed for patch-level models to observe the patch grouping of each model (Fig. 4). t-SNE analysis of the color-model led to grouping according to nucleus size (Fig. 4a). As a result of RI-model analysis, grouping according to nucleus size was still observed, but the RI model’s group boundaries were ambiguous when compared to those of the color-model (Fig. 4b). In many cases, viewing the detailed structure of the patch was difficult when the sample was on the boundary region in the t-SNE plot of the RI model. However, when both the color- and RI-models were used together, the benign and malignant groups were more distinctly separated (Fig. 4c).

## Discussion

In this study, a combination of RI image data and color Papanicolaou-stained image data improved the accuracy of MLA for diagnosing cancer using thyroid FNAB specimens. The classification results of the MLA using color Papanicolaou-stained images were highly dependent on the size of the nucleus, but those of the MLA using RI images were less dependent on nucleus size and were affected by information around the nuclear membrane. The final algorithm using data from both types of images together distinguished thyroid cell clusters from benign thyroid nodules and PTC with 100% accuracy.

MLA has shown superior diagnostic performance using images of thyroid FNAB specimens when a convolutional neural network (CNN) architecture was adopted, which is effective for image analysis^7,8,12,13^. Guan et al.^13^ studied a CNN-based MLA for classifying hematoxylin–eosin-stained FNAB specimens of benign thyroid nodule and PTC (TBSRTC II, V and VI). A total of 887 fragmented color images were used in this study, which were cropped from 279 images taken using a digital camera attached to a brightfield microscope. The trained algorithm exhibited 97.7% accuracy for distinguishing between 128 test images of benign and malignant nodules. Range et al.^8^ used MLA to classify Papanicolaou-stained FNAB specimens of broader spectrum thyroid nodules (TBSRTC II–VI). They used 916 color images obtained using a whole slide scanner. The trained MLA distinguished malignant from benign nodules with high accuracy (90.8%), comparable to that of a pathologist. Similarly, a CNN-based MLA performed well in our study, exhibiting high-accuracy patch-level classification (97.3%) and cluster-level classification (99.0%), using only color Papanicolaou-stained images.

However, given that the purpose of FNAB is to determine whether to operate on thyroid nodules, it must not only exhibit high overall accuracy, but also minimize serious misclassification, such as classification of an obvious malignancy as benign or that of an overtly benign nodule as a malignancy. In Guan’s study, MLA misclassified some cases that a pathologist classified as obviously benign as a malignancy. Similarly, in Range’s study, MLA misclassified some clearly benign nodules as malignant or misclassified a malignant nodule that was indicated for surgery as benign^8^. These issues are problematic because they can lead to an erroneous treatment plan for patients who would receive proper treatment if they underwent the current standard care. We studied nodules with relatively distinct benign or malignant characteristics (TBSRTC II, V, and VI). Our findings that RI data improved the accuracy of MLA in these nodules have important clinical significance since these indicate a potential reduction in the aforementioned serious misclassification.

Guan et al.^13^ suggested that the significant misclassifications of MLA for the thyroid FNAB specimens could be related to the nucleus size. In their study, the cells in false-positive cases showed large nuclei with a high mean pixel color information similar to malignant cells, but the pathologist determined that these cells had a typically benign morphology. The authors interpreted that the classification of MLA was based on the size and staining of the nucleus, but not on the shape. Furthermore, in our results, MLA based on color images showed limitations in accurately classifying benign thyroid cells with a large nucleus or malignant thyroid cells with a small nucleus because the size of the nucleus was the main feature required for classification. However, MLA classification based on the RI image was less affected by nucleus size. This suggests that RI images for can compensate for the limitations of MLA using color images for FNAB specimens whose nuclear size is not typical for benign or malignant cells.

Further results from analyses to explain the models suggest that RI-image based MLA uses the structure and shape of the nucleus for classification. In addition to the algorithm being activated mainly for large nuclei in color images, the algorithm was activated not only by large nuclei but also by nuclei with a clear structure in RI images. The certainty of the MLA classification results was proportional to the detail of the information around the nuclear membrane when based on RI images, but not when based on color images. Detailed nuclear structures, such as nuclear membrane irregularity and micronucleoli are important indicators of thyroid cancer diagnosis^26^. Thus, the accuracy of MLA classification can be improved when such information is incorporated.

Another potential strength of RI images is the integration of information of a wide vertical space. In a thyroid cytology specimen, cells are scattered over a wide vertical space (i.e. multiple z-plains) rather than over a plane. A single layer (z-plain) 2D image cannot address this vertical spread, and information from out-of-focus cells is likely to be lost or distorted. In contrast, in the RI image obtained through ODT, cells located in different Z-plains are in focus simultaneously. In our study, MLA based on color images showed a false positive result for some out-of-focus patches, whereas MLA based on RI image showed a true negative result for the same image patches (data not shown). However, the out-of-focus area is only a part of the color images, and the use of multiple z-plane images did not improve the accuracy of MLA when compared to the use of a single z-plane image in a previous study^8^. Therefore, it is unclear whether the aforementioned factor significantly affects the accuracy of MLA.

This study has certain limitations. Despite the large number of sample measurements, this study was performed in a single center and could not cover all conditions of specimens that could exist in real clinical environments. ODT provides optimal RI imaging in un-manipulated living cells^27^, but we obtained RI images from chromatically stained cells. Staining acted as an extrinsic noise or artifact in the RI images, which reduced the accuracy of MLA. Further study is required to determine the effect of staining on the outcomes. Finally, up to 30% of FNABs may have “indeterminate” cytopathology (TBSRTC III and IV). This study targeted specimen characteristic of benign or malignant thyroid nodules (TBSRTC II, V, and VI), and therefore, the currently trained algorithm cannot be directly applied to TBSRTC III and IV specimens without relevant training.

To investigate the complementary nature of RI images and color images, a 2D MIP image was generated by projecting the 3D RI image along the z-axis, thereby excluding the influence of dimensionality. Previous studies in the field of cell classification have demonstrated improved performance when using 3D RI images compared to 2D images^28,29^. Although our research did not incorporate 3D images due to the specific research objectives, we plan to expand our investigations in future studies by incorporating 3D RI images and other 3D imaging modalities.

In this study, we demonstrated the efficacy of multiplexing of RI with standard brightfield imaging using a single ODT platform for MLA-based classification of benign and malignant thyroid FNABs. Multiplexed ODT showed promise for the development of a more accurate classification of thyroid FNABs while reducing the inherent uncertainty and error observed in the current diagnostic standards. Thus, an ODT-based MLA may potentially contribute to an improved cost-effective and rapid point-of-care management of thyroid malignancies.

## Supplementary Information

Supplementary Information.

## Data Availability

The datasets used and/or analysed during the current study available from the corresponding authors on reasonable request.

## References

[1] Haugen BR (2016). 2015 American thyroid association management guidelines for adult patients with thyroid nodules and differentiated thyroid cancer: The American thyroid association guidelines task force on thyroid nodules and differentiated thyroid cancer. Thyroid.

[2] Fitzmaurice C (2015). The global burden of cancer 2013. JAMA Oncol..

[3] Vaccarella S (2015). The impact of diagnostic changes on the rise in thyroid cancer incidence: A population-based study in selected high-resource countries. Thyroid.

[4] Udelsman R, Zhang Y (2014). The epidemic of thyroid cancer in the United States: The role of endocrinologists and ultrasounds. Thyroid.

[5] Lee YK (2019). Changes in the diagnostic efficiency of thyroid fine-needle aspiration biopsy during the era of increased thyroid cancer screening in Korea. Cancer Res. Treat..

[6] Jiang Y, Yang M, Wang S, Li X, Sun Y (2020). Emerging role of deep learning-based artificial intelligence in tumor pathology. Cancer Commun. (Lond.).

[7] Kezlarian B, Lin O (2021). Artificial intelligence in thyroid fine needle aspiration biopsies. Acta Cytol..

[8] Elliott Range DD (2020). Application of a machine learning algorithm to predict malignancy in thyroid cytopathology. Cancer Cytopathol..

[9] Savala R, Dey P, Gupta N (2018). Artificial neural network model to distinguish follicular adenoma from follicular carcinoma on fine needle aspiration of thyroid. Diagn. Cytopathol..

[10] Gilshtein H, Mekel M, Malkin L, Ben-Izhak O, Sabo E (2017). Computerized cytometry and wavelet analysis of follicular lesions for detecting malignancy: A pilot study in thyroid cytology. Surgery.

[11] Landau MS, Pantanowitz L (2019). Artificial intelligence in cytopathology: A review of the literature and overview of commercial landscape. J. Am. Soc. Cytopathol..

[12] Dey P (2021). The emerging role of deep learning in cytology. Cytopathology.

[13] Guan Q (2019). Deep convolutional neural network VGG-16 model for differential diagnosing of papillary thyroid carcinomas in cytological images: A pilot study. J. Cancer.

[14] Sabottke CF (2022). The effect of image resolution on deep learning in radiology. Radiol. Artif. Intell..

[15] Schiff L, Migliori B, Chen Y (2022). Integrating deep learning and unbiased automated high-content screening to identify complex disease signatures in human fibroblasts. Nat. Commun..

[16] Park Y, Depeursinge C, Popescu G (2018). Quantitative phase imaging in biomedicine. Nat. Photon..

[17] Esposito M (2021). TGF-β-induced DACT1 biomolecular condensates repress Wnt signalling to promote bone metastasis. Nat. Cell Biol..

[18] Jo Y (2021). Label-free multiplexed microtomography of endogenous subcellular dynamics using generalizable deep learning. Nat. Cell Biol..

[19] Pham HV, Pantanowitz L, Liu Y (2016). Quantitative phase imaging to improve the diagnostic accuracy of urine cytology. Cancer Cytopathol..

[20] Boustany NN, Boppart SA, Backman V (2010). Microscopic imaging and spectroscopy with scattered light. Annu. Rev. Biomed. Eng..

[21] Hunter M (2006). Tissue self-affinity and polarized light scattering in the born approximation: A new model for precancer detection. Phys. Rev. Lett..

[22] Chantziantoniou N, Donnelly AD, Mukherjee M, Boon ME, Austin RM (2017). Inception and development of the papanicolaou stain method. Acta Cytol..

[23] Barer R (1953). Determination of dry mass, thickness, solid and water concentration in living cells. Nature.

[24] Popescu G (2008). Optical imaging of cell mass and growth dynamics. Am. J. Physiol. Cell Physiol..

[25] Kim, D.* et al.* Holotomography: Refractive index as an intrinsic imaging contrast for 3-D label-free live cell imaging. In *Advanced Imaging and Bio Techniques for Convergence Science* 211–238 (Springer, 2021). 10.1007/978-981-33-6064-8_10 33834439

[26] Cibas ES, Ali SZ (2017). The 2017 Bethesda system for reporting thyroid cytopathology. Thyroid.

[27] Popescu G, Park Y (2015). Quantitative phase imaging in biomedicine. J. Biomed. Opt..

[28] Kim G, Ahn D, Kang M (2022). Rapid species identification of pathogenic bacteria from a minute quantity exploiting three-dimensional quantitative phase imaging and artificial neural network. Light Sci. Appl..

[29] Ryu D (2021). Label-free white blood cell classification using refractive index tomography and deep learning. BME Front..

