# Supplementary material for: Machine-learning-based diagnosis of thyroid fine-needle aspiration biopsy synergistically by Papanicolaou staining and refractive index distribution
Source: Sci Rep. 2023 Jun 17;13:9847. doi: 10.1038/s41598-023-36951-2 (PMC10276805; doi:10.1038/s41598-023-36951-2)
Supplement: Supplementary file 1 — Supplementary Information. [file 41598_2023_36951_MOESM1_ESM.docx]

**Supplementary Text**

**1. Model architecture**

We use CNN-model called DenseNet-169 model for patch-level model and XGBoost classifier for cluster-level model. Due to the different image channels in color and 2D RI images (three channels for color images and a single channel for 2D RI images), we trained two separate models.

To diversify the training images using a limited amount of data, data augmentation techniques (randomly cropping to a smaller size than the original image and randomly flipping the image horizontally) were used for training both the color images and 2D RI images, where elastic transformation was additionally used for 2D RI images. All images were normalized with mean = (0.4914, 0.4822, 0.4465) and standard deviation = (0.2023, 0.1994, 0.2010) for three-channel color images and mean = 0.445 and standard deviation = 0.269 for single channel 2D RI images.

**2. Training details**

**Label generation**

We adopted a cluster-level approach to determine malignancy of the sample. Subsequently, all patches derived from the same cluster were assigned the same label, effectively creating a pseudo-label for each patch. It is important to note that when considering patch-level analysis alone, there may be instances where the assigned label differs from the actual malignancy status of the corresponding sample. However, as the patch-level model was primarily trained as a feature extraction tool for cluster-level classification, the label noise resulting from the application of cluster-level labeling to patch-level data did not significantly impact the final cluster-level classification outcomes.

Furthermore, in order to validate the patch-level classification tendencies of our model, we consulted clinical experts. Their assessment confirmed the accuracy of the model’s patch-level malignancy predictions. In fact, when there were discrepancies between the pseudo-label and the patch-level model’s output, the model’s output consistently demonstrated a higher level of accuracy (data not shown).

**Patch level prediction**

Patch-level CNN model $f_{\theta}$with the parameter $\theta$ maps the input image $\mathbf{x}$ to the probability of malignancy, indicating a value close to 0 as negative (benign) and 1 as positive (malignant). The original architecture consists of blocks of convolution layers for extracting latent features and a fully connected MLP layer for predicting the probability of malignancy using aggregated latent features. We replace the last fully connected classification layer with the neural bootstrapping classifier. It enables to perform bootstrapping without extra resources for training and inference, and the efficiency of training models with small dataset sizes (or small number of training data) has been observed. For each image, five bootstrapping samples are made, and the final prediction is made by averaging the prediction of bootstrapping samples.

Training patch level model, the error is computed between output of the model $\hat{y}=f_{\theta}(\mathbf{x})$ and the ground truth $y$ using binary cross entropy loss $l$ ($l=-\frac{1}{N}\sum_{i=0}^{N} y_{i}\cdot\log(\hat{y_{i}})+ (1-y_{i})\cdot log(1-\hat{y_{i}})$). By averaging errors computed over mini-batch sized of 32, we use Adam optimizer with parameters $\beta_{1}=0.5$ and $\beta_{2}=0.999$ and a learning rate of 0.002 with the cosine-annealing learning rate scheduler.

As of binary classification task on patch level prediction, we set a threshold of 0.5 on output probability deciding benign or malignant.

**Cluster feature extraction**

Using the patch-level trained models, we can extend patch predictions into cluster level. Since patches are extracted to contain at least one nucleus via half-size sliding window, the probability map (or heatmap) of cluster is made by averaging patch prediction scores over overlapping regions.

The prediction for color and RI images is made by averaging the prediction scores on 50% region at the center of prediction map since most of the nuclei are aggregated at the center of cluster image.

Making cluster-level prediction, we exploit cluster (global) and patch (local) features using both color and RI images. We take average the center region of cluster heatmap made by malignancy probability of patches (cluster-wise feature) on each color and 2D RI clusters. Also, patch-wise prediction results for the color and 2D RI patches located in the center region are compared (patch-wise feature): (1) how many patches predicted the same results, (2) how many patches predicted at least one model as malignant, and (3) how many patches predict differently or provided different results.

**Cluster level prediction**

For cluster level prediction using both color and 2D RI images, we use two cluster-level features by averaging 50% centered region of color and 2D RI cluster heatmaps and three patch-wise comparison features on the XGBoost classifier model. XGBoost is an ensemble model based on decision trees with a gradient boosting algorithm that minimizes the errors of sequential data. We set the number of gradient boosted tress as 200, which is equivalent to the number of epochs in training neural networks, with a depth of 6.

While training the XGBoost model, we use both training and validation cluster data due to the limited number of training clusters, 1,249 clusters in total. Although it is difficult to use different modal features while predicting each color and 2D RI cluster, we use the center region of the cluster heatmap as the prediction of the cluster.

Since cluster have different sizes ranges from 300 (pixel) to 2000 (pixel), there exist clusters where only few patches are sampled. Clusters with less than five patches are extracted does not provide enough patch-level features. Therefore, we consider 30 clusters with less than five sampled patches as an outlier to prevent bias on patch-level features on training cluster-level model.

Using both cluster-wise and patch-size features of color and 2D RI images, our model correctly predicts the malignancy of clusters. This shows different models that using both color and 2D RI images together, we can effectively predict the malignancy of clusters.

We have tested on different MLA models for cluster prediction. We can confirm that using both color and RI images are complementary providing robust performances agnostic to models. The cluster-level performances on Random Forest classifier, Support Vector Machine classifier and Multi-layer Perceptron with one hidden layer is summarized in Supplementary Table 3.

**3. Visualization**

**Visualization of model prediction**

To interpret the prediction result, we used Grad-CAM. Unlike CAM which uses only the probabilities and the last feature vector, Grad-CAM additionally uses the gradient values passed to the last convolution layer, emphasizing the local features of the malignant points.

**Visualization of the feature space**

For training patch level models, patches are extracted from the cluster by applying the patching rule. We used t-distributed stochastic neighbor embedding (t-SNE) over a 1669-dimensional feature vector obtained from the last convolution layer of patch-level model for reducing high-dimensional features into two dimensions on Figure 6. Each path with a different ground truth has a different color bar, and the colors varies depending on the prediction probability. For each number of clusters denoted on the scatter plot, the left side shows the patch examples of benign clusters, whereas the patches from malignant clusters are shown on the right side.

**4. Brenner Gradient**

The concept of the Brenner gradient was introduced by J. F. Brenner et al.^26^. It is a simple yet rapid edge detector, which measures the difference between a pixel and its neighbor, as follows:

$$B=\sum_{i=1}^{width} \sum_{j=1}^{height} \left[ s\left( i, j \right)-s\left( i+m, j \right) \right]^{2}$$

where *m* is the distance between two pixels. A higher Brenner gradient *B* denotes a greater difference between neighboring pixels, which means more edges in the image.

To determine whether the RI image shows more details in the nucleus than the color image, the Brenner gradient for each patch was calculated while restricting the target area to only near the nuclei mask. The mask for the nucleic region was generated using k-means clustering. The resulting Brenner gradient value was normalized by the area of the mask to compare the complexity of nucleic pixel values.

**Supplementary Table 1.**

| Label | Benign | Malignancy | | | Total |
| --- | --- | --- | --- | --- | --- |
| Bethesda classification | II  (n = 88) | V  (n = 5) | VI  (n = 31) | V + VI  (n = 36) | II + V + VI  (n=124) |
| Age (years) | 54.5 | 43.4 | 48.7 | 47.9 | 52.6 |
| Sex |  |  |  |  |  |
| Male (n, %) | 14, 15.9% | 3, 60.0% | 9, 29.0% | 12, 33.3% | 26, 21.0% |
| Female (n, %) | 74, 84.1% | 2, 40.0% | 22, 71.0% | 24, 66.7% | 98, 79.0% |
| Total number of clusters (n) | 1,128 | 44 | 363 | 407 | 1,535 |
| Number of clusters per slide (median, IQR) | 15.5 / 12 | 6 / 8 | 13 / 8.5 | 13 / 10 | 14 / 12 |
| Total number of image patches (n) | 14,515 | 733 | 8,110 | 8,843 | 23,358 |
| Number of image patches per slide (median, IQR) | 134 / 182 | 78 / 68 | 201 / 313.5 | 182.5 / 326 | 157 / 188.75 |
| Number of image patches per cluster (median, IQR) | 10 / 9 | 14 / 8.25 | 17 / 16 | 16 / 15 | 13 / 11 |

Description of the dataset of the total number of classes and the number of clusters and patches extracted from the slide and cluster, respectively. Labels indicating benign for Bethesda classification II and malignancy for Bethesda classification V (suspicious for malignancy) and VI (malignancy) clusters.

**Supplementary Table 2.**

| **Index** | **Terminology** | **Abbrevation** | **Descrpition** |
| --- | --- | --- | --- |
| 1 | Color brightfield of Papanicolaou staining image | Color image | One of the most widely used multi-color staining technique in cytology. It has advantages in clear staining in nuclear chromatin, counterstaining cytoplasm, and transparency. |
| 2 | Refractive index image | RI image | An image generated by 3D refractive index distributions. |
| 3 | Fine-Needle Aspiration Biopsy | FNAB | A biopsy that collects tissue penatraing fine needle under the skin. This is mostly done on breat, thyroid gland and lymph nodes. |
| 4 | Quantitative Phase Imaging | QPI | An imaging technique that measures and quantifies the phase information of light waves passing through a transparent or semi-transparent samples. |
| 5 | Optical Diffraction Tomography | ODT | A 3D label-free imaging tool that captures quantitative RI distribution within semi-transparent samples. |
| 6 | Machine Learning Algorithm | MLA | An algorithm, or a mathematical model that captures the underlying patterns from data and predict using learned parameters. |
| 7 | Convolutional Neural Network | CNN | A type of deep learning model that uses convolution layers to captures information under cross-correlated kernel. It is mostly used in computer vision area. |
| 8 | Class Activation Map | CAM | A technique that visualizes the relevance of pixel regions and prediction results using neural network model. |
| 9 | t-distributed Stochastic Neighbor Embedding | t-SNE | A statistical model that maps high-dimensional data into low dimenional map using distribution over similarity of features. Minimizing distributions, it enables to cluster similar features close together in low dimension space. |
| 10 | Receiver Operating Characteristic curve | ROC curve | A graphical plot that depicts the classification model performance as discriminative threshold changes. |

Table explaining the terminology, abbreviation and their explanations used in the paper.

**Supplementary Table 3.**

|  | **Accuracy** | **Sensitivity** | **Specificity** | **PPV** | **NPV** |
| --- | --- | --- | --- | --- | --- |
| **XGBoost** | 1.0 | 1.0 | 1.0 | 1.0 | 1.0 |
| **Random Forest** | 1.0 | 1.0 | 1.0 | 1.0 | 1.0 |
| **Support  Vector Machine** | 1.0 | 1.0 | 1.0 | 1.0 | 1.0 |
| **Multi-layer Perceptron** | 1.0 | 1.0 | 1.0 | 1.0 | 1.0 |

Cluster-level classification performances on different MLA algorithms: XGBoost, Random Forest, Support Vector Machine and Multi-layer Perceptron.

**Supplementary Figure 1.**


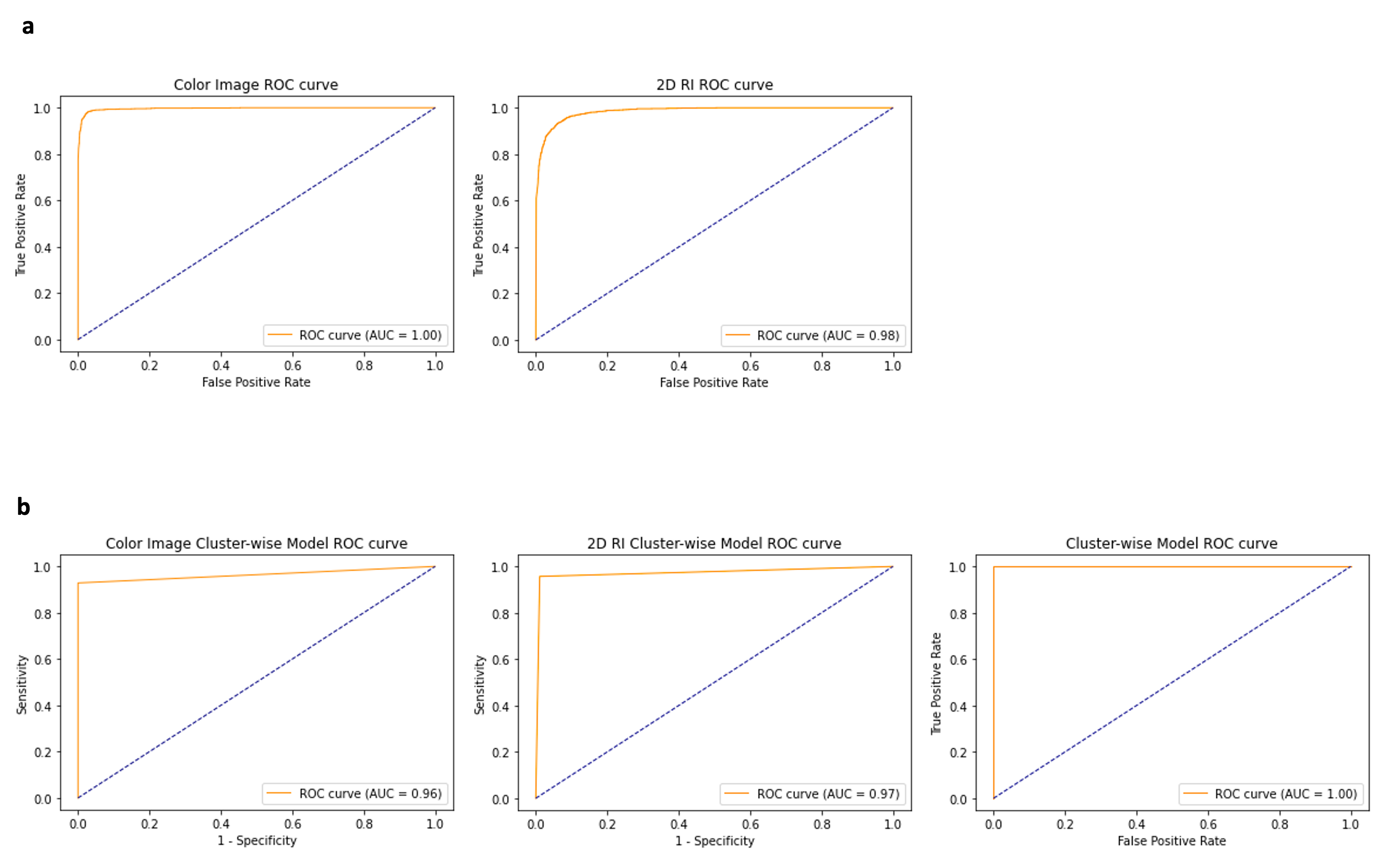


**(a)** The ROC curves of the patch-level model for color images and 2D RI images and (**b)** the ROC curves of the cluster-level model for color images and 2D RI images and the combined result.

**Supplementary Figure 2.**


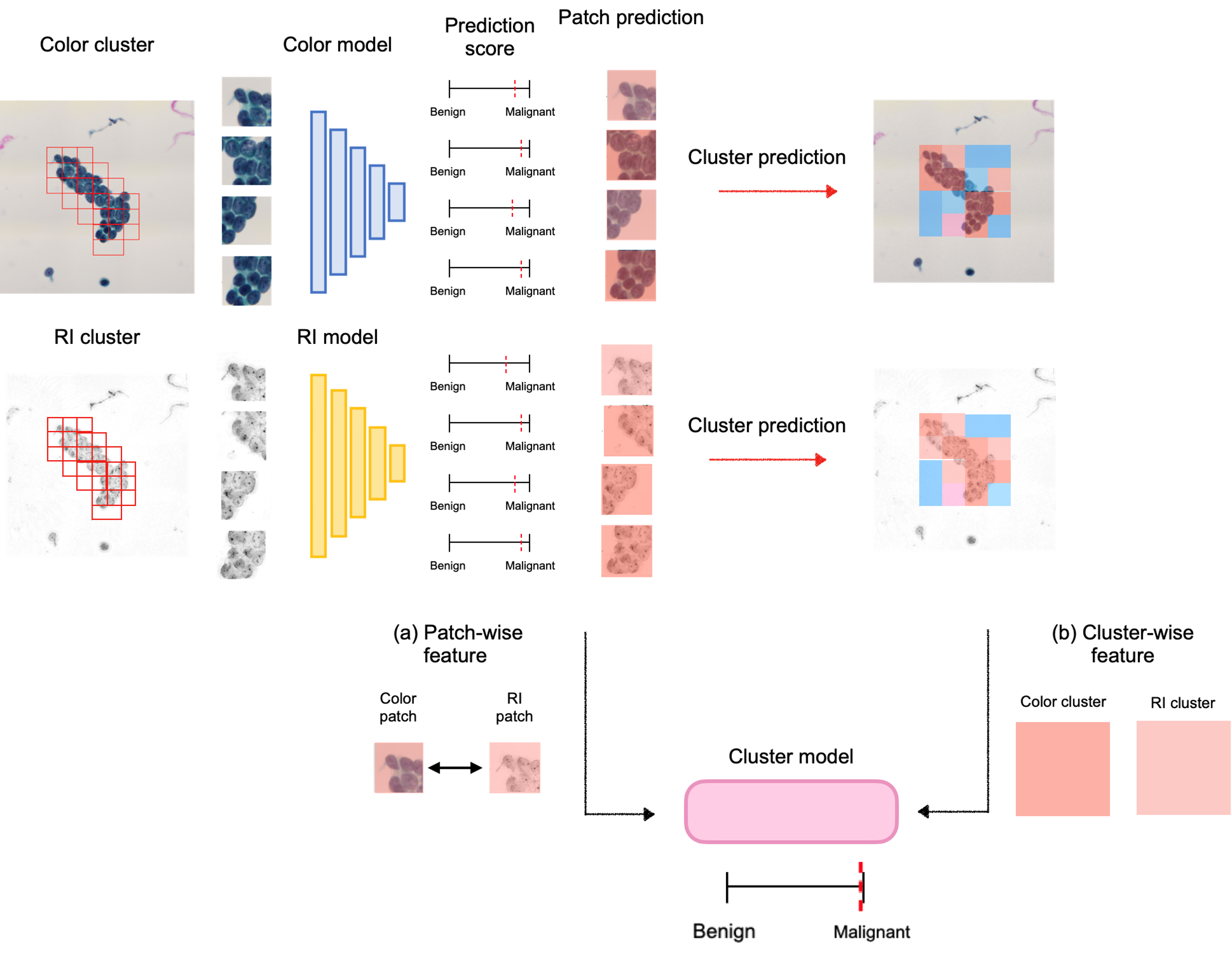


Detail description of performing cluster-level prediction. For each color and RI cluster, same number of patches are extracted from same pixel position. Using patch-level model, we can compute the prediction score ranges 0 (benign) to 1 (malignant). We expand this result into clusters, generating prediction map of 50% region of clusters. (a) patch-wise features are calculated via comparing corresponding color and RI patches (how many patches have same prediction results, different prediction results, and predicted as malignant at least one model). (b) cluster-wise features are computed by averaging 50% region of cluster prediction map for each color and RI clusters.

**Supplementary Figure 3.**

**
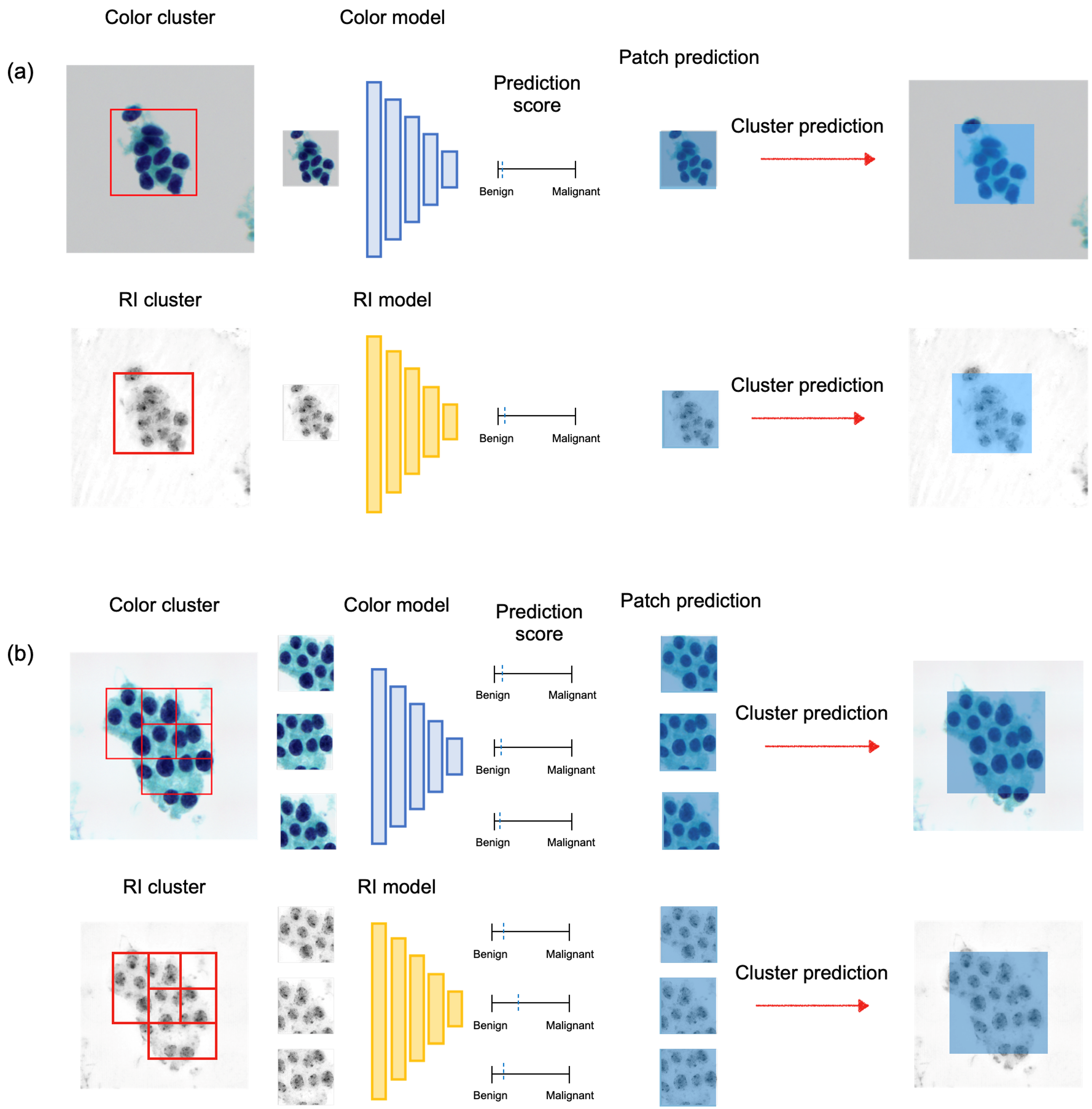
**

Excluded clusters while predicting cluster level when **(a)** only one patch is extracted due to small size of cluster image and (**b)** only few patches (less than five patches) are extracted. Note that patches extracted from same cluster follows the same label.
